# Antibacterial activity of *Tribulus* *terrestris* and its synergistic effect with Capsella bursa-pastoris and *Glycyrrhiza* *glabra* against oral pathogens: an in-vitro study

**Published:** 2015

**Authors:** Saman Soleimanpour, Fereshteh Sadat Sedighinia, Akbar Safipour Afshar, Reza Zarif, Kiarash Ghazvini

**Affiliations:** 1 *Antimicrobial resistance Research Center, School of medicine, Mashhad University of Medical Sciences, Mashhad, Iran *; 2 *Department of Biology, Neyshabur Branch, Islamic Azad University, Neyshabur, Iran*

**Keywords:** *Antibacterial Activity*, *Tribulusterrestris*, *Glycyrrhizaglabra*, *Capsella bursa-pastoris*, *Oral Pathogens*

## Abstract

**Objective:**

In this study, antimicrobial activities of an ethanol extract of *Tribulus* *terrestris* aloneand in combination with *Capsella bursa-pastoris *and *Glycyrrhiza* *glabra *were examined in vitro against six pathogens namely *Streptococcus mutans, Streptococcus sanguis, Actinomyces* *viscosus, Enterococcus faecalis Staphylococcus aureus,* and *Escherichia coli*.

**Materials and methods:**

Antibacterial activities of the extracts were examined using disc and well diffusion methods and the minimum inhibitory concentration (MIC) and minimum bactericidal concentration (MBC) of ethanol extracts were determined against these microorganisms using agar and broth dilution methods. Chlorhexidine was used as positive control.

**Results:**

*Tribulus* *terrestris* extract exhibited good antibacterial activity against all bacteria. Antibacterial activity of mixed extract was evaluated and exhibited that mixed extract was more effective against all bacteria than any of the cases alone which indicates the synergistic effect between these three extracts (p˂0.05). No strain showed resistance against these extracts. In agar dilution, *Tribulus* *terrestris* exhibited MIC values ranging from 35.0 to 20.0 mg/ml and mixed extract showed MIC values ranging from 12.5 to 5.0 mg/ml. The results of broth dilution method were consistent with the findings of the agar dilution method.

**Conclusion:**

This *in-vitro* study was a preliminary evaluation of antibacterial activity of the plants. It provided scientific evidence to support uses of T. terrestris and its mixture with C. bursa-pastoris and *G. glabra* for the treatment of oral infections. *In-vivo* studies are also required to better evaluate the effect of these extracts.

## Introduction

Dental caries and periodontal diseases are still considered as two main common dental pathologies affecting humankind and inflict a costly burden to health care services around the world, especially in developing countries (Singh et al., 2007 ▶; Poole, 2001 ▶). Oral infections and dental caries are common oral bacterial pathology caused by a biofilm consisting of oral microbiota present on the tooth surface. Dental plaque is formed by the colonization and accumulation of oral microorganisms such as *Streptococcus mutans*, *Streptococcus sanguis*, and *Actinomyces viscosus* on teeth surface (Singh et al., 2007 ▶). Mechanical removal of the dental plaque by antiseptic agents such as chlorhexidine is the most efficient procedure in caries prevention. The undesirable side effects of certain antibiotics and resistance against them have limited the preventive measures. As a result, this situation has forced scientists to look for new antimicrobial substances from various sources such as medicinal plants (Cai and Wu, 1996 ▶). Over the past years, several studies on the antimicrobial properties of plants have been studied in different regions of the world and Iran (Sedighinia et al., 2012 ▶; Soleimanpour et al., 2013 ▶; Janovska et al., 2003 ▶; FazlyBazzaz et al., 2003 ▶). *Tribulus terrestris*, *Glycyrrhiza glabra*, and *Capsella bursa-pastoris* are three plants native to Khorasan (North of Iran) and have been used in Iranian traditional medicine as antiseptic and antimicrobial remedies for the treatment of many different health problems. *Glycyrrhiza glabra* and *Capsela bursa-pastoris* were examined and observed in previous studies which showed that they both had anti-bacterial effects on oral pathogens (Sedighinia et al., 2012 ▶; Soleimanpour et al., 2013 ▶). *Tribulus terrestris* L. is a member of the *Zygophyllaceae* family distributed in warm regions of the world. *T. terrestris* is used in folk medicine as tonic, aphrodisiac, analgesic, astringent, stomachic, anti-hypertensive, diuretic, lithon-triptic, and urinary anti-infective (Kianbakht and Jahaniani, 2003 ▶). The main components of *T. terrestris* are saponins, diosgenins, alkaloids, and amides (Yan et al., 1996 ▶; Borke et al., 1992 ▶). Although there are some studies on antimicrobial activity and anti-urinary infection of *T. terrestris *but there is no research about oral pathogens. In this study, we evaluated the antibacterial activity of *Tribulus terrestris *and its mixture with *G. glabra* and *C. bursa-pastoris* against oral pathogens.

## Materials and Methods

**Plant material**

*Source, collection and identification*

Total parts of *C. bursa-pastoris* and roots of *G. glabra* were collected from Garineh, a village near Neyshabour, and fruit of *T. terrestris* was collected from Noghondar, a village near Mashhad, Khorasan province (north of Iran), during summer 2011. A voucher specimen for each plant was prepared and identified at the Research Institute of Plant Sciences Herbarium, Ferdowsi University of Mashhad, Iran. 

*Extract* *preparation*

The plant material (Fruit of *T. terrestris* (25 g), different parts of *C. bursa-pastoris *(25 g), and roots of *G. glabra* (25 g) were air dried at 25 **°**C and ground in a mechanical grinder to a fine powder. The powdered material (250 g) was extracted with 300 ml of ethanol at room temperature for 72 h. Extracts were filtered and the solvent was evaporated on the rotary vacuum evaporator (Heidolphlaborota 4000, Germany) under reduced pressure at 40 **°**C (Sedighinia et al., 2012 ▶; Soleimanpour et al., 2013 ▶; More et al., 2008 ▶). For preparation of mixed extract including *T. terrestris, G. glabra, *and *C. bursa-pastoris*, equal amounts (2 mL) of each extract (100 mg/mL) was thoroughly mixed in a sterile tube. Therefore, the concentration of each extract was 33.33 mg/ml in the mixed extract. The extracts were further dried at room temperature after which they were subjected to antimicrobial tests.

**Antibacterial activity**

*Microbial strains*

The microorganisms used in this study included *Streptococcus mutans *(PTCC 1683), *Streptococcus sanguis *(PTCC 1449), *Actinomyces viscosus *(PTCC 1202), and *Enterococcus faecalis *(ATCC 29212) as oral pathogens as well as *Staphylococcus aureus* (ATCC 25923) and *Escherichia coli *(ATCC 29922) as controls. The bacterial strains were cultured in brain-heart infusion (BHI) medim (Difco, MI, USA) under anaerobic condition in an anaerobic jar with Anaerocult A (Merk SA (Pty) Ltd), 37 **°**C for 72 h and subculturing was done twice weekly. Suspensions of the test organisms were prepared by picking colonies from appropriately incubated agar cultures to sterile broth, to match a McFarland 0.5 turbidity standard (approximately 1.5x10^8^ CFU/mL) (McFarland, 1907 ▶).

*Disk diffusion and well diffusion methods*

Microbial growth inhibitory potential of the *T. terrestris* and mixed extracts were determined using the agar disk diffusion method as described by CLSI (CLSI, 2009 ▶). *T. terrestris* and mixed extract were diluted to concentrations ranging from 100 to 3.125 mg/ml and chlorhexidine 0.2% mouthwash (Donya Behdasht, Tehran, Iran) with concentrations ranging from 0.0625 up to 2 mg/ml and distilled water were used as positive and negative controls, respectively. Twenty microtiter of the plant extracts and chlorhexidine concentration were transferred onto sterile filter papers (6.4 mm diameter). Each Mueller-Hinton agar (with 5% sheep blood) was uniformly seeded by means of sterile swab dipped in the suspension and streaked on the agar plate surface. The plates were then incubated at 37 **°**C for 48 h anaerobically. All tests were performed in triplicate and zones of inhibition were measured. The agar-well diffusion method was performed as prescribed by NCCLS as well. Wells of 5 mm in diameter were punched in the Mueller-Hinton agar (with 5% sheep blood) using a sterile cork-borer about 2 cm apart. Approximately, 20 μl of the extracts were dropped into each well which filled them respectively to fullness. The rest of the process was performed as described previously (NCCLS, 2012 ▶). 

**Determination of minimum inhibitory concentration (MIC) and minimum bactericidal concentration (MBC)**

*Macro broth dilution method*

The minimum inhibitory concentration (MIC) of the *T. terrestris* and mixed extracts were determined according to methods described by CLSI 2006. *T. terrestris* and mixed extracts were diluted to concentrations ranging from 100 to 0.78 mg/mL in 1 mL Mueller-Hinton broth. Four to five isolated colonies from an overnight culture were selected and diluted in broth to achieve a turbidity equivalent to a 0.5 McFarland standard (10^8^ CFU/mL). This dilution was further diluted 1:100 (10^6^ CFU/mL) with broth and then 0.5 mL of bacterial broth suspension was added to each tube (CLSI, 2006 ▶). Control tubes with no bacterial inoculation were simultaneously maintained. Tubes were incubated anaerobically at 37 **°**C for 24 hours. The lowest concentration of the extracts that produced no visible bacterial growth (turbidity) was recorded as the MIC (CLSI, 2006 ▶). To estimate the MIC of the extracts more precisely and for confirmation of the results, a more precise concentration in agar dilution method was used.

*Agar dilution method*

Agar dilution assay was used to test the susceptibility of the microorganisms to the *T. terrestris* and mixed extracts at different concentrations, as recommended by the Clinical Laboratory Standards Institute (CLSI). Serial dilutions of *T. terrestris* and mixed extracts were prepared in plates according to the standard procedure. After solidification, the plates were incubated at 37 **°**C for 2 h in order to dry the agar surface. The assay plates were estimated to have 50, 35, 30, 25, 20, 15, 12.5, 10, 5, 6.25, 3.125, 2.5, and 1.25 mg/ml of active extracts. Inocula were applied to agar surfaces in 1 µl spots, giving approximately 1.5×10^5^ cfu per spot. Plates without added extract were inoculated as viability controls and uninoculated media were also included to confirm sterility. All plates were inverted and incubated appropriately for 48 to 72 h in anaerobic condition. The MIC was considered as the lowest concentration of extract, which caused a marked inhibition in growth as compared to the growth control. This extract was tested in triplicate vs. each organism (three separate inoculums preparations on three different days) (CLSI, 2009 ▶).

**Statistical analysis**

Results are presented as the means ± SD of at least three replicates. The Student t-test was used for statistical analyses of the difference noted. P values of 0.05 or less were considered statistically significant.

## Results

In vitro antibacterial activity of *T. terrestris* extract and its mixture with *G. glabra* and *Capsella bursa-pastoris* extracts and their potency were quantitatively and qualitatively assessed by determining the inhibition zone diameter and MIC as given in Tables 1-6.

**Table 1:** Antimicrobial activities of *T. terrestris* against oral microorganisms controlled with zones of inhibition in millimeter using disk diffusion method (mean±SD).

**Plant extract**	**Concentration** **mg/mL**	***S. mutans***	***S. sanguis***	***A. viscosus***	***E. faecalis***	***S. aureus***	***E. coli***
***T. terrestris***	3.125	12.9 ± 0.9	-	8.6 ± 1	9 ± 0.0	9 ± 0.0	8.4 ± 0.0
	6.25	13.6 ± 0.4	6 ± 0.0	13.5 ± 0.7	10.5 ± 0.5	10.3 ± 0.5	9.1 ± 0.8
	12.5	19.5 ± 0.7	9 ± 0.7	15.4 ± 0.8	14.1 ± 0.5	15 ± 0.7	14.9 ± 0.5
	25	21.8 ± 1.3	11 ± 0.0	19 ± 1.4	15.6 ± 0.5	18 ± 0.3	17.4 ± 0.5
	50	22.7 ± 0.99	14.3 ± 0.7	22.9 ± 1.2	18 ± 1	21.6 ± 1.1	21.3 ± 0.5
	100	24.6 ± 0.66	16.2 ± 0.6	25.2 ± 1	21 ± 1	25.4 ± 1.3	24.2 ± 0.6
**Negative Control**		-	-	-	-	-	-

-: No inhibition zone, These results showed that antibacterial activity of this extract was significantly greater than negative control (p<0.05).

**Table 2:** Antimicrobial activities of mixed extract (*T. terrestris, G. glabra* and *C. bursa-pastoris*) against oral microorganisms controlled with zones of inhibition in millimeter using disk diffusion method (mean±SD).

**Plant extract**	**Concentration** **mg/mL**	***S. mutans***	***S. sanguis***	***A. viscosus***	***E. faecalis***	***S. aureus***	***E. coli***
**Mixed extract**	3.125	18 ± 0.0	15.4 ± 0.8	12.2 ± 0.5	12.4 ± 0.8	18.6 ± 0.5	17.2 ± 0.5
	6.25	21.8 ± 0.4	19 ± 0.0	14 ± 0.0	14 ± 0.0	21.8 ± 0.4	20 ± 0.0
	12.5	25 ± 0.0	22.2 ± 0.6	17.8 ± 0.4	18.6 ± 0.5	25 ± 0.0	22 ± 0.0
	25	26.6 ± 0.4	23 ± 0.0	20.4 ± 0.8	20.8 ± 0.4	26.2 ± 0.5	23.8 ± 0.4
	50	29 ± 0.0	25.8 ± 0.4	25 ± 0.0	21 ± 0.0	29.2 ± 0.5	25.8 ± 0.4
	100	31.4 ± 0.8	28.2 ± 0.6	27.4 ± 0.8	23.8 ± 0.4	31.8 ± 0.4	30 ± 0.0
**Negative control**		-	-	-	-	-	-

-: No inhibition zone, These results showed that antibacterial activity of this extract was significantly greater than negative control (p<0.05).

**Table 3:** Antimicrobial activities of *T. terrestris* against oral microorganisms and controlled with zones of inhibition in millimeter using well diffusion method (mean±SD).

**Plant extract**	**Concentration** **mg/mL**	***S. mutans***	***S. sanguis***	***A. viscosus***	***E. faecalis***	***S. aureus***	***E. coli***
***T. terrestris***	3.125	8 ± 0.0	-	-	8 ± 0.0	10.1 ± 0.2	9 ± 0.0
	6.25	8.4 ± 0.0	-	13.2 ± 0.2	12 ± 0.3	12 ± 0.0	13.2 ± 0.2
	12.5	16 ± 0.3	10.1 ± 0.2	16 ± 0.0	17.3 ± 0.2	16 ± 0.0	16 ± 0.0
	25	19.1 ± 0.8	14.4 ± 0.0	18.2 ± 0.3	20 ± 0.0	19.2 ± 0.2	17.4 ± 0.5
	50	21.8 ± 0.3	18 ± 0.0	21.4 ± 0.5	23.8 ± 0.3	21 ± 0.0	20.2 ± 0.4
	100	22.2 ± 0.2	20.4 ± 0.5	23.8 ± 0.4	25.6 ± 0.5	25.8 ± 1	23 ± 0.0
**Negative Control**		-	-	-	-	-	-

-: No inhibition zone

The results obtained by above-mentioned method confirmed that antibacterial activity of this extract was significantly greater than negative control (p<0.05)

**Table 4:** Antimicrobial activities of mixed extract (*T. terrestris, G. glabra,* and *C. bursa-pastoris*) against oral microorganisms and controlled with zones of inhibition in millimeter using well diffusion method (mean±SD).

**Plant extract**	**Concentration** **mg/mL**	***S. mutans***	***S. sanguis***	***A. viscosus***	***E. faecalis***	***S. aureus***	***E. coli***
**Mixed extract**	100	31.8 ± 0.3	30 ± 0.0	29 ± 0.0	25 ± 0.0	32 ± 1	31.2 ± 0.3
	50	29.4 ± 0.5	27 ± 1	27.2 ± 0.2	23.3 ± 0.2	30 ± 0.0	27.4 ± 0.8
	25	27.4 ± 0.5	25 ± 1	24.1 ± 0.2	20 ± 0.0	28.2 ± 0.3	25 ± 0.0
	12.5	25.4 ± 0.0	23.4 ± 0.8	20.6 ± 0.5	19 ± 1	27.8 ± 0.4	23 ± 0.0
	6.25	21.4 ± 0.5	20 ± 0.0	15.8 ± 0.3	16.4 ± 0.5	22 ± 0.0	20 ± 1
	3.125	18 ± 0.0	17 ± 0.0	14 ± 1	13.3 ± 0.2	19 ± 0.0	17.4 ± 0.5
**Negative control**		-	-	-	-	-	-

-: No inhibition zone

The results obtained by above-mentioned method confirmed that antibacterial activity of this extract was significantly greater than negative control (p< 0.05).

**Table 5:** Mean MIC (mg/mL) results of *T. terrestris* and mixed extracts (*T. terrestris, G. glabra,* and *C. bursa-pastoris*) on oral microorganisms and controls in agar dilution method.

**Plant extract**	***S. mutans***	***S. sanguis***	***A. viscosus***	***E. faecalis***	***S. aureus***	***E. coli***
***T. terrestris*** ** extract**	20	35	35	35	25	25
**Mixed extract **	5	12.5	10	10	6.25	6.25

**Table 6:** Mean MIC and MBC (mg/mL) results of *T. terrestris* and mixed extracts (*T. terrestris, G. glabra,* and *C. bursa-pastoris*) on oral microorganisms and controls in broth dilution method

**Plant extract**		***S. mutans***	***S. sanguis***	***A. viscosus***	***E. faecalis***	***S. aureus***	***E. coli***
***T. terrestris***	**MIC**	25	50	50	50	25	25
	**MBC**	25	50	50	50	25	25
**Mixed extract **	**MIC**	6.25	12.5	6.25	6.25	6.25	6.25
	**MBC**	6.25	12.5	6.25	6.25	6.25	6.25

**Table 7:** Antimicrobial activities of the chlorhexidine against oral microorganisms and controlled with zones of inhibition in millimeter using disk diffusion method (mean±SD).

**Plant extract**	**Concentration** **mg/mL**	***S. mutans***	***S. sanguis***	***A. viscosus***	***E. faecalis***	***S. aureus***	***E. coli***
**Chlorhexidin**	0.625	9.5 ± 0.5	-	8.2 ± 0.2	7 ± 0.0	10 ± 1	-
	0.125	12 ± 0.0	10.5 ± 0.0	10 ± 0.0	9.2 ± 0.2	13.5 ± 0.5	12.2 ± 0.2
	0.25	15 ± 0.0	13.7 ± 0.99	12 ± 0.5	12 ± 0.0	17.4 ± 0.7	16 ± 0.0
	0.5	18 ± 0.0	14.2 ± 0.2	14 ± 1	17.5 ± 0.5	20 ± 0.0	18 ± 0.0
	1	23 ± 0.0	15.5 ± 0.0	17 ± 0.0	24.2 ± 0.1	24.5 ± 0.5	21.2 ± 0.0
	2	25 ± 0.0	17 ± 0.0	24 ± 0.0	25 ± 0.0	25.2 ± 0.2	23 ± 0.0
**Negative Control**		-	-	-	-	-	-

-: No inhibition zone

These results showed that antibacterial activity of chlorhexidine, a well-known antibacterial agent, was not significantly greater than other extracts (p>0.05).

**Table 8:** Antimicrobial activity of the chlorhexidine against oral microorganisms controlled with zones of inhibition in millimeter using well diffusion method (mean±SD).

**Plant extract**	**Concentration** **mg/mL**	***S. mutans***	***S. sanguis***	***A. viscosus***	***E. faecalis***	***S. aureus***	***E. coli***
**Chlorhexidine**	0.625	9.5 ± 0.5	-	9 ± 0.0	-	-	-
	0.125	13 ± 0.0	10.7 ± 0.4	10.2 ± 0.2	10 ± 0.0	13.5 ± 0.0	12.5 ± 0.0
	0.25	15.2 ± 0.2	12.2 ± 0.2	13.6 ± 0.5	14.2 ± 0.2	16.5 ± 0.5	17 ± 0.0
	0.5	20 ± 0.0	14.2 ± 0.2	15.5 ± 0.0	18 ± 0.0	22 ± 0.0	21 ± 0.0
	1	24 ± 0.0	16.7 ± 0.4	19 ± 0.0	24.2 ± 0.1	24 ± 0.5	22 ± 0.0
	2	27.5 ± 0.5	21 ± 0.0	23 ± 0.0	27 ± 0.0	27.2 ± 0.2	24.2 ± 0.2
**Negative Control**		-	-	-	-	-	-

-: No inhibition zone

These results showed that antibacterial activity of chlorhexidine, a well-known antibacterial agent, was not significantly greater than other extracts (p>0.05).

**Table 9:** Mean MIC (mg/mL) results of chlorhexidine extract on oral microorganisms and controls in agar and broth dilution method.

**Agar dilution method**	***S. mutans***	***S. sanguis***	***A. viscosus***	***E. faecalis***	***S. aureus***	***E. coli***
						
**MIC**	0.0625	0.0625	0.0625	0.0625	0.0625	0.125
**Broth dilution method**						
**MIC**	0.0625	0.0625	0.0625	0.0625	0.0625	0.125
**MBC**	0.125	0.125	0.0625	0.125	0.125	0.125

## Discussion

Recently, several antibiotics and antiseptic agents such as chlorhexidine and cetylpyridinium chloride have been used widely in dentistry to inhibit bacterial growth (Renton-Harper et al, 1996 ▶). Several side effects of these substances and the development of antimicrobial resistant pathogens have become an ever-increasing therapeutic problem. Reasons in question and many others justify further research and development of natural antimicrobial agent targeting specific oral pathogens while being safe for the host (Cai and Wu, 1996 ▶). In the recent decade, antimicrobial activity of plants in different areas of the world and Iran has also been studied (Janovska et al., 2003 ▶; Fazly Bazzaz et al., 2003 ▶; Javadnia et al., 2009 ▶). All these studies show that plant species with anti-microbial activity is very diverse around the world and also in Iran. In one study, the methanol extracts of 306 plants of 52 families obtained from Northeast of Iran, were tested for their antimicrobial activity. Among 171 extracts with antimicrobial effects, 10 extracts had the highest activity (Fazly Bazzaz et al., 2003 ▶). We evaluated the antibacterial activity of three extracts from these plants including *T. terretris* (present study), *G. glabra* (Sedighinia et al., 2012 ▶), *C. bursa-pastoris *(Soleimanpour et al., 2013 ▶), and mixture of them against oral pathogens. In previous studies, *Glycyrrhiza glabra* and *Capsela bursa-pastoris* were examined and the results showed that both of them had anti-bacterial effects on oral pathogens. The ethanolic extract of *T. terrestris* had promising MIC value against all oral bacteria especially *S. mutans* and exhibited the highest MIC value against *S. sanguis, A. viscosus, *and* E.* *faecalis*. Therefore, the present study supports the idea that *T. terrestris* extract might be useful as an antibacterial agent against oral pathogens. The findings propose that *T. terrestris* can inhibit the growth of *Streptococcus mutans, Actinomyces viscosus,* *Streptococcus sanguis, *and *Enterococcus* *faecalis. *Therapeutic effects of *T. terrestris* extract on oral diseases such as dental caries and periodontal diseases have been shown in some other studies. In one study, it was shown that ethanol extract of *T. terrestris* inhibited the growth and acid production of *S. mutans* (Hong-Keun et al., 2011 ▶). Other studies in Iraq (Ahmed et al., 2009 ▶), Turkey (Abbasoglu and Tosun, 1994 ▶ ), India, and Iran (Kianbakht et al., 2003 ▶) have shown that this extract has a good antibacterial activity against gram positive bacteria such as *S. aureus* and *E. faecalis* and gram negative bacteria such as *E. coli*. It indicates that there is a broad spectrum of antibiotic compounds or simply general metabolic toxins in the plant. In this study, for the first time, antibacterial activity of *T. terrestris* against *A. viscosus *and* S. sanguis *was confirmed and shown that the ethanolic extract of this plant had promising MIC value against all oral bacteria especially *S. mutans.* The antimicrobial activity of *T. terrestris, C. bursa-pastoris,* and *G. glabra* extracts in this study has been shown in some other studies separately but antibacterial effects of mixed extract of these plants on oral pathogens has not been studied yet. In the present study, antibacterial activity of mixed extract including *T. terrestris*, *C. bursa-pastoris*, and *G. glabra* was evaluated and shown that mixed extract was more effective against all bacteria than any of the cases alone that indicates the synergistic effect between these three extracts. Therefore, this *in-vitro* study provides scientific evidence to support uses of *T. terrestris* and its mixture with *C. bursa-pastoris* and *G. glabra* for the treatment of oral infections and suggests them as a candidate that may help us to control dental caries and periodontal diseases. The effects of these extracts may be more beneficial if they are incorporated in toothpaste, mouthwash, and dental products to reduce plaque and dental caries. Further studies are required to better evaluate the effect of these extracts and to isolate the bioactive compounds responsible for the observed activities.

## References

[B1] Abbasoglu U, Tosun F (1994). Antimicrobial Activity of Tribulus terrestris L. Growing in Turkey. Hacettepe Universitesi Eczacilik Fakultesi Dergisi.

[B2] Ahmed A Hussain, Abbas A Mohammed, Heba H Ibrahim, Amir H. Abbas (2009). Study the Biological Activities of Tribulus Terrestris Extract. World Acad Sci Eng Technol.

[B3] Borke CA, Stevens GR, Garriqan MJ (1992). Locomotor Effects in Sheeps of Alkaloids Identified in Australian Tribulus terrestris. Aust Vet J.

[B4] Cai L, Wu CD (1996). Compounds from Syzygium aromaticum possessing growth inhibitory activity against oral pathogens. J Nat Prod.

[B5] CLSI (Clinical and Laboratory Standards Institute) (2009). Performance standards for antimicrobial disk susceptibility test. Approved standard M02-A10.

[B6] CLSI (2006). Methods for Dilution Antimicrobial Susceptibility Tests for Bacteria That Grow Aerobically. Approved Standard M7-A7.

[B7] CLSI (2009). Methods for antimicrobial susceptibility testing of anaerobic bacteria. Approved standard M11-A7.

[B8] FazlyBazzaz BS, Haririzadeh G (2003). Screening of Iranian plants for antimicrobial activity. Pharmaceut Biol.

[B9] Hong-Keun Oh, Soo Jeong Park, Hae Dalma Moon, Seung Hwan Jun, Na-Young Choi, Yong-Ouk You (2011). Tribulus terrestris inhibits caries-inducing properties of Streptococcus mutans. J Med Plants Res.

[B10] Janovska D, Kubikova K, Kokoska L (2003). Screening for Antimicrobial Activity of Some Medicinal Plants Species of Traditional Chinese Medicine. Czech J Food Sci.

[B11] Javadnia K, Miri R, Assadollahi M, Gholami M, Ghaderi (2009). Screening of Selected Plants Growing in Iran for Antimicrobial. Iran J Sci Tech Trans.

[B12] Kianbakht S, & Jahaniani F (2003). Evaluation of Antibacterial Activity of Tribulus terrestris L. Growing in Iran. Iran J Pharmacol Therap.

[B13] McFarland J (1907). The nephelometer: an instrument for estimating the number of bacteria in suspensions for calculating the opsonic index and vaccines. J Am Med Assoc.

[B14] More G, Tshikalange TE, Lall N, Botha F, Meyer JJM (2008). Antimicrobial activity of medicinal plants against oral microorganisms. J Ethnopharmacology.

[B15] NCCLS (National Committee for Clinical Laboratory Standards) (2000). Methods for dilution: Antimicrobial susceptibility test for bacteria that grow aerobically. M-7-A5.

[B16] Poole K (2001). Overcoming antimicrobial resistance by targeting resistance mechanisms. J Pharma Pharmacol.

[B17] Renton-Harper P, Addy M, Moran J, Doherty FM, Newcombe RG (1996). A comparison of chlorhexidine, cetylpyridinium chloride, triclosan, and C31G mouthrinse products for plaque inhibition. J Periodonto.

[B18] Sedighinia F, Safipour Afshar A, Soleimanpour S, Zarif R, Asili J, Ghazvini J (2012). Antibacterial activity of Glycyrrhiza glabra against oral pathogens: an in vitro stud. Avicenna J Phytomed.

[B19] Singh J, Kumar A, Budhiraja S, Hooda A (2007). Ethnomedicine: use in dental caries. Braz J Oral Sci.

[B20] Soleimanpour S, Sedighinia F, Safipour Afshar A, Zarif R, Asili J, Ghazvini K (2013). Synergistic Antibacterial Activity of Capsella bursa-pastoris and Glycyrrhiza glabra Against Oral Pathogens. Jundishapur J Microbiol.

[B21] Yan W, Ohtani K, Kasai R, Yamasaki K (1996). Steroidal Saponins from Fruits of Tribulus terrestris. Phytochemistry.

